# Radiomics and Qualitative Features From Multiparametric MRI Predict Molecular Subtypes in Patients With Lower-Grade Glioma

**DOI:** 10.3389/fonc.2021.756828

**Published:** 2022-01-21

**Authors:** Chen Sun, Liyuan Fan, Wenqing Wang, Weiwei Wang, Lei Liu, Wenchao Duan, Dongling Pei, Yunbo Zhan, Haibiao Zhao, Tao Sun, Zhen Liu, Xuanke Hong, Xiangxiang Wang, Yu Guo, Wencai Li, Jingliang Cheng, Zhicheng Li, Xianzhi Liu, Zhenyu Zhang, Jing Yan

**Affiliations:** ^1^ Department of Neurosurgery, The First Affiliated Hospital of Zhengzhou University, Zhengzhou, China; ^2^ Department of Neurology, The First Affiliated Hospital of Zhengzhou University, Zhengzhou, China; ^3^ Department of Pathology, The First Affiliated Hospital of Zhengzhou University, Zhengzhou, China; ^4^ Institute of Biomedical and Health Engineering, Shenzhen Institutes of Advanced Technology, Chinese Academy of Sciences, Shenzhen, China; ^5^ Department of MRI, The First Affiliated Hospital of Zhengzhou University, Zhengzhou, China

**Keywords:** lower-grade glioma, radiomics, Visually Accessible Rembrandt Images, molecular subtypes, machine learning

## Abstract

**Background:**

Isocitrate dehydrogenase (IDH) mutation and 1p19q codeletion status have been identified as significant markers for therapy and prognosis in lower-grade glioma (LGG). The current study aimed to construct a combined machine learning-based model for predicting the molecular subtypes of LGG, including (1) IDH wild-type astrocytoma (IDHwt), (2) IDH mutant and 1p19q non-codeleted astrocytoma (IDHmut-noncodel), and (3) IDH-mutant and 1p19q codeleted oligodendroglioma (IDHmut-codel), based on multiparametric magnetic resonance imaging (MRI) radiomics, qualitative features, and clinical factors.

**Methods:**

A total of 335 patients with LGG (WHO grade II/III) were retrospectively enrolled. The sum of 5,929 radiomics features were extracted from multiparametric MRI. Selected robust, non-redundant, and relevant features were used to construct a random forest model based on a training cohort (n = 269) and evaluated on a testing cohort (n = 66). Meanwhile, preoperative MRIs of all patients were scored in accordance with Visually Accessible Rembrandt Images (VASARI) annotations and T2-fluid attenuated inversion recovery (T2-FLAIR) mismatch sign. By combining radiomics features, qualitative features (VASARI annotations and T2-FLAIR mismatch signs), and clinical factors, a combined prediction model for the molecular subtypes of LGG was built.

**Results:**

The 17-feature radiomics model achieved area under the curve (AUC) values of 0.6557, 0.6830, and 0.7579 for IDHwt, IDHmut-noncodel, and IDHmut-codel, respectively, in the testing cohort. Incorporating qualitative features and clinical factors into the radiomics model resulted in improved AUCs of 0.8623, 0.8056, and 0.8036 for IDHwt, IDHmut-noncodel, and IDHmut-codel, with balanced accuracies of 0.8924, 0.8066, and 0.8095, respectively.

**Conclusion:**

The combined machine learning algorithm can provide a method to non-invasively predict the molecular subtypes of LGG preoperatively with excellent predictive performance.

## Introduction

Diffuse lower-grade glioma [LGG, World Health Organization (WHO) grades II and III] is a primary brain tumor that originates from glial or precursor cells and presents as a heterogeneous disease (1). The 2016 WHO classification divides LGG into three molecular subtypes based on isocitrate dehydrogenase (IDH) mutation and 1p19q codeletion status (1): IDH wild-type (IDHwt) (2), IDH mutants with euploid 1p19q (IDHmut-noncodel), and (3) IDH mutants carrying 1p19q codeletion (IDHmut-codel) (2, 3). The role of molecular diagnosis in the classification of central nervous system (CNS) tumors was further clarified in the 2021 WHO criteria (4).

Previous research has shown that IDHmut-noncodel glioma patients had lower overall survival than IDHmut-codel patients, although both subtypes had significantly higher overall survival than patients with IDHwt gliomas (5). Additionally, LGG with IDH mutation or 1p19q codeletion is more sensitive to radiation and chemotherapy than IDHwt LGG (6–8). Biopsy is the gold standard for confirming molecular biomarkers. However, the invasive biopsy-based approach carries a certain risk of neurological deficit and morbidity. Therefore, a non-invasive, low-cost method able to predict the molecular subtypes of LGG at an early stage could provide better guidance for risk–benefit assessment and individualized treatment decision-making.

Traditional radiographic assessment of LGG most commonly relies on visual evaluation (9). Visually Accessible Rembrandt Images (VASARI) annotations on preoperative magnetic resonance imaging (MRI) have been reported to predict IDH mutation and 1p19q codeletion in gliomas with good performance (10, 11). Patel et al. reported that T2-weighted-fluid-attenuated inversion recovery (T2-FLAIR) mismatch sign (T2 hyperintense signal and FLAIR hypointense signal aside from a hyperintense peripheral rim) is an important imaging biomarker for discriminating the IDH and 1p19q status of LGG (12). However, these human-recognized imaging features cannot embrace all the multidimensional and subtle patterns presented by MRI.

Radiomics is a novel method for the high-throughput extraction of quantitative features from a specified region of interest from images (13). Recently, machine learning-based radiomics analysis has been successfully applied to quantify radiographic features for identifying image biomarkers with the capability to predict genotypes and the clinical outcomes of various tumors (9, 14). Previous studies have leveraged radiomics analysis to predict IDH mutation and 1p19q codeletion status in gliomas (15–18). However, these studies only focused on single biomarkers or provided a multilevel binary classifier, which is not straightforward and has limited clinical application. Moreover, previous studies frequently utilized conventional MR sequences such as contrast-enhanced T1-weighted imaging (CE-T1WI), T2-weighted imaging (T2WI), and FLAIR imaging (15, 18). However, apparent diffusion coefficient (ADC) maps calculated from diffusion-weighted imaging (DWI) have been reported to play a vital role in glioma classification (19).

In this study, we constructed a machine learning-based combined model with multiple classifications based on clinical factors, radiomics, and qualitative features from multiparametric MRI, including T1WI, CE-T1WI, T2WI, FLAIR, and ADC imaging to predict IDH mutation and 1p19q codeletion status in LGG. We aimed to develop a more convenient approach to preoperatively predict the molecular subtypes of LGG.

## Materials and Methods

### Patients

Ethics approval for this study was obtained from the Human Scientific Ethics Committee of the First Affiliated Hospital of Zhengzhou University (No. 2019-KY-176). Among the 604 patients receiving craniotomy for tumor resection and pathologically diagnosed with LGG in the Department of Neurosurgery, the First Affiliated Hospital of Zhengzhou University, from July 2009 to July 2019, 335 were further selected according to the following criteria: (1) adult patients (age ≥18 years); (2) histopathological diagnosis of primary grade II/III glioma; (3) availability of IDH and 1p/19q status; (4) availability of preoperative multiparametric MRI, including axial T1WI, CE-T1WI, T2WI, FLAIR, and ADC imaging; and (5) availability of sufficient image quality without significant artifacts, as determined by neuroradiologists and neurosurgeons. The selection procedure is shown in **Supplementary Figure S1**. Clinical factors (gender and age) were obtained from the medical record system.

### MRI Acquisition

All patients were examined on either 1.5- or 3.0-T clinical MR scanners from Siemens, Philips, or GE Healthcare. The brain MRI protocol included the following sequences: (a) axial and sagittal T1WI, (b) axial T2WI, (c) axial FLAIR imaging, and (d) DWI and the corresponding ADC maps generated with the software incorporated into the MRI unit; and (e) axial, sagittal, and coronal CE-T1WI obtained immediately after intravenous administration of a gadolinium-based contrast agent. Details of the MR machines and sequence parameters are provided in the **Supplementary Material**.

### Image Preprocessing and Tumor Delineation

**Figure 1:**
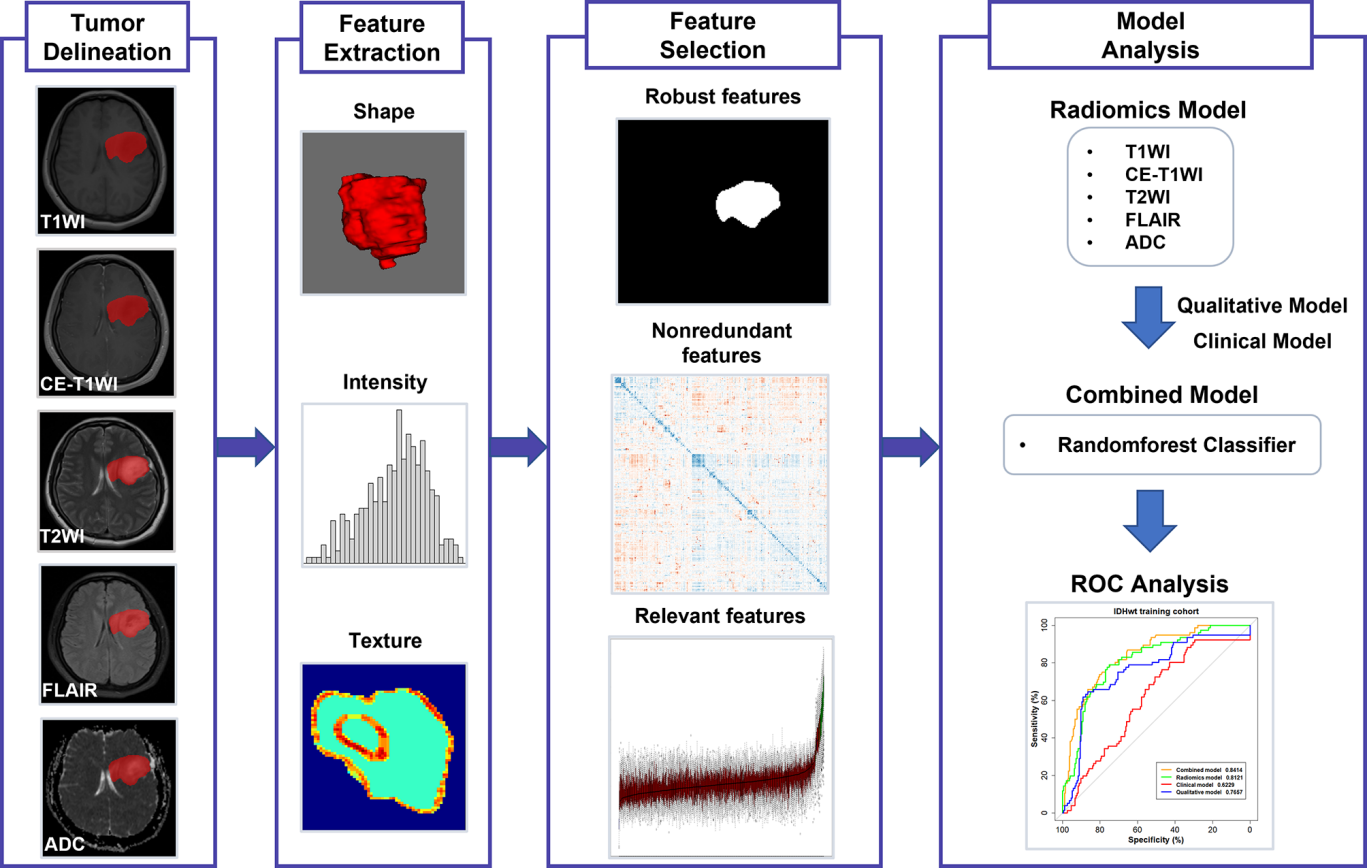
The workflow of this study.

The workflow of this study is shown in **Figure 1**. Image preprocessing was performed to standardize the images. First, the N4 bias field correction was applied to remove any low frequency intensity non-uniformity (20). After all voxels were isotropically resampled into 1 × 1 × 1 mm^3^ using trilinear interpolation, multiparametric MRI samples were co-registered to the corresponding CE-T1WI using a rigid transformation. Histogram matching was used to normalize signal intensity. A batch-effect correction tool ComBat (21) was used to remove scanner and site effects. The three-dimensional volume of interest (VOI) of tumor contours was manually delineated using the open-source software ITK-SNAP (www.itk-snap.org) by a neuroradiologist (JY with 11 years of experience), primarily on axial FLAIR images where T2WI and CE-T1WI were used to cross-check the extension of the tumor and fine-tune the tumor contour. According to BraTS subvolume convention (22), the VOIs were delineated as the whole tumor region, including the edema, enhancing core, the non-enhancing core, and the necrotic/cystic core. The VOIs were re-delineated in 60 cases (IDHwt, n = 20; IDHmut-noncodel, n = 20; IDHmut-codel, n = 20), which were randomly selected among the included patients by a senior neuroradiologist (JC with 20 years of experience). The segmented VOI was then overlaid with the co-registered resampled T1WI, CE-T1WI, T2WI, FLAIR, and ADC images. Neuroradiologists were blinded to clinical, pathological, and molecular data.

### Radiomics Feature Extraction

All radiomics features were extracted using Pyradiomics extractor. Three groups of features were extracted: shape features, first-order intensity features, and higher-order texture features. Texture features were extracted using five different methods, including the gray-level co-occurrence matrix (GLCM), gray-level run length matrix (GLRLM), gray-level size zone matrix (GLSZM), gray-level dependence matrix (GLDM), and neighborhood gray-tone difference matrix (NGTDM). Two filters [wavelet transform and Laplacian of Gaussian (LoG) with four sigma levels (2.0, 3.0, 4.0, and 5.0)] were enabled in the extracted intensity and texture features. The extracted features are summarized in **Supplementary Table S1**. The extracted features were consistent with the Imaging Biomarker Standardization Initiative (IBSI) (23).

### Qualitative Feature Review: VASARI Scores and T2-FLAIR Mismatch Sign

We selected 25 semantic descriptors of imaging features from VASARI annotations based on preoperative MRI: f1, tumor location; f2, side of lesion center; f3, eloquent brain; f4, enhancement quality; f5, proportion enhancing; f6, proportion non-contrast-enhancing tumor; f7, proportion necrosis; f8, cysts; f9, multifocal or multicentric; f10, T1/FLAIR ratio; f11, thickness of enhancing margin; f12, definition of the enhancing margin; f13, definition of the non-enhancing margin; f14, proportion of edema; f15, edema crosses midline; f16, hemorrhage; f17, diffusion characteristics; f18, pial invasion; f19, ependymal invasion; f20, cortical involvement; f21, deep white matter invasion; f22, non-enhancing tumor crosses midline; f23, enhancing tumor crosses midline; f24, satellites; and f25, calvarial remodeling. The exact description of all these features can be found in The Cancer Imaging Archive VASARI research project webpage (https://wiki.cancerimagingarchive.net/display/Public/VASARI+Research+Project). Each tumor was independently scored by a neuroradiologist (JY) and a neurosurgeon (ZZ with 11 years of experience), according to the VASARI scoring system based on the five MR sequences using ITK-SNAP. Furthermore, the T2-FLAIR mismatch sign, which has been defined as an easily detectable imaging sign on routine clinical MRI studies for the diagnosis of IDHmut-noncodel gliomas (24), was also assessed. Any disagreement between the two raters was resolved through discussion and consensus. A qualitative feature dataset was obtained by combining the VASARI features and T2-FLAIR mismatch signs.

### Feature Selection

For radiomics features, all features were standardized using z-score normalization. First, the stability of the extracted features was evaluated by interobserver reproducibility of the two image readers. Intraclass correlation coefficient (ICC) values were calculated for each feature of the 60 patients. Features with ICC value ≥0.90 were selected in this study. The correlation coefficient between each pair of features was calculated to eliminate redundancy. For feature pairs with correlation coefficients >0.75, the feature with the worst univariate predictive power (smaller Mann–Whitney U-test p-value) was removed. Based on the qualitative feature dataset and the remaining robust and non-redundant radiomics feature data set, the R package Boruta (25) was used to select the optimal all relevant features. Boruta is a random forest-based all-relevant feature selection wrapper algorithm that iteratively compares the importance of original features with the importance of artificially added random features, progressively removing irrelevant features. The most important features of the qualitative and radiomics feature datasets were obtained.

### Machine Learning Classification

First, a radiomics model based on selected radiomics features was constructed using the R package randomForest to classify the three molecular subtypes. Then, a combined model with selected radiomics features, selected qualitative features, and clinical factors was constructed using the random forest algorithm. For comparison, a clinical model based on clinical factors (gender and age) and a qualitative model based on VASARI features and T2-FLAIR mismatch signs were constructed using the same algorithm. Besides, a radiomics model without ADC sequence was also built. Gini index was used as importance measure (26).

### Statistical Analysis

All statistical analyses were performed using the R software (version 4.0.5, http://www.Rproject.org). Statistical significance was set at p < 0.05. The patients in this study were randomly divided into a training cohort and a testing cohort at a ratio of 4:1, where the distribution of the clinical characteristics was balanced. Differences in gender, age, and molecular subgroups between the training and testing cohorts were assessed using the t-test or χ^2^ test. Differences in patient characteristics across the three molecular subtypes were assessed using the Kruskal–Wallis test. In the training cohort, 10-fold cross-validation was applied to optimize the parameters of random forest classifiers in all four classification models (combined model, radiomics model, qualitative model, and clinical model). The testing cohort was used for the final model evaluation. The classification performance (one specific class versus all other classes) was assessed using receiver operating characteristic (ROC) analysis according to the area under the curve (AUC), balanced accuracy, sensitivity, and specificity. The maximum value of the Youden index (sensitivity + specificity − 1) was chosen as the optimal cutoff for each binary classification. All indices were calculated for both training and testing cohorts. The AUCs were statistically compared between different classifiers using DeLong analysis.

## Results

### Patient Characteristics

A total of 335 patients were included in the current study according to the selection criteria. The patients were divided into training (n = 269) and testing (n = 66) cohorts. There were no significant differences in clinical factors and molecular subtypes between the training and testing cohorts, as shown in **Table 1**. The distribution of patient characteristics across the three molecular subtypes is shown in **Supplementary Table S2**.

**Table 1:** Distribution of patient characteristics in the training cohort and testing cohort.

Characteristic	Overall (n = 335)	Training Cohort (n = 269)	Testing Cohort (n = 66)	p-Value
Gender				0.9417
Male	189 (53.24%)	151 (53.94%)	38 (64.20%)	
Female	146 (43.58%)	118 (46.06%)	28 (35.80%)	
Age(year)*	44.93 ± 12.47	44.59 ± 12.27	46.27 ± 13.24	0.3519
Molecular subtypes				0.7837
IDHwt	94 (28.06%)	76(29.13%)	18(24.69%)	
IDHmut-noncodel	110 (32.84%)	86 (31.89%)	24 (35.80%)	
IDHmut-codel	131 (39.10%)	107 (38.98%)	24 (39.51%)	

Unless otherwise noted, data are numbers of patients, with percentages in parentheses.

*Data are means ± standard deviations.

### Radiomics Features Selection

We extracted 1,197 features from each sequence ([14 shape features, 234 intensity features (18 were from original images, 72 were from LoG images, and 144 were from wavelet images), 949 texture features (73 original texture features, 292 LoG texture features, and 584 wavelet features)]. In total, 5,929 radiomic features were extracted from the five MRI sequences for each patient. After the robustness tests, 3,103 out of 5,929 features remained. After redundancy reduction, 335 features were selected for the subsequent analyses. The heat maps of the correlation coefficients of both the 3,103 features and the selected 335 features are shown in **Supplementary Figure S2**. After the Boruta feature selection, the 17 most important features for an optimal model fit were finally selected, including 11 texture features and 6 intensity features, as shown in **Table 2**. The results of the Boruta feature selection are shown in **Supplementary Figure S3**, where the boxplots of the importance of all features fed to Boruta are shown. The univariate association of each selected feature with the molecular subtype was significant (false discovery rate adjusted, p < 0.001). There were no significant differences in selected 17 radiomics features between different scanners and different field strengths (ANOVA test, p > 0.05), as shown in **Supplementary Figures S4** and **S5**, respectively.

**Table 2:** Selected radiomics features for predicting the molecular subtypes of lower-grade glioma patients.

No.	Selected Features	Type	Sequence	Filter	pFDR
f_1_	Interquartile range	Intensity	ADC	Original	<0.001
f_2_	Skewness	Intensity	ADC	Original	<0.001
f_3_	NGTDM Complexity	Texture	ADC	log-sigma-3-0-mm	<0.001
f_4_	GLCM ClusterShade	Texture	ADC	log-sigma-5-0-mm	<0.001
f_5_	GLRLM RunVariance	Texture	ADC	log-sigma-5-0-mm	<0.001
f_6_	Median	Intensity	ADC	Wavelet. HLL	<0.001
f_7_	GLCM ClusterShade	Texture	ADC	Wavelet. HLL	<0.001
f_8_	GLCM Imc1	Texture	FLAIR	log-sigma-3-0-mm	<0.001
f_9_	GLRLM RunVariance	Texture	FLAIR	log-sigma-4-0-mm	<0.001
f_10_	Skewness	Intensity	FLAIR	Wavelet. LHL	<0.001
f_11_	GLRLM GrayLevelNonUniformityNormalized	Texture	T1WI	Wavelet. LLH	<0.001
f_12_	GLRLM RunVariance	Texture	T1WI	Wavelet. LHH	<0.001
f_13_	GLCM SumEntropy	Texture	T1WI	Wavelet. HLL	<0.001
f_14_	GLDM LargeDependenceEmphasis	Texture	T1WI	Wavelet. HLL	<0.001
f_15_	Skewness	Intensity	T1WI	Wavelet. LLL	<0.001
f_16_	GLRLM LongRunHighGrayLevelEmphasi	Texture	T1WI	Wavelet. LLL	<0.001
f_17_	Skewness	Intensity	CE-T1WI	Original	<0.001

H and L were high- and low-pass filters in wavelet transform, respectively. pFDR is short for false discovery rate-adjusted p-value.

### Qualitative Feature Selection

The Boruta algorithm revealed 10 qualitative features that were significantly associated with the three molecular subtypes. The results of the Boruta feature selection are shown in **Supplementary Figure S6**. Finally, nine VASARI features and T2-FLAIR mismatch sign were selected to build a qualitative model: f1, tumor location (V1); f4, enhancement quality (V2); f5, proportion enhancing (V3); f6, proportion non-contrast enhancing tumor (V4); f11, thickness of enhancing margin (V5); f13, definition of the non-enhancing margin (V6); f20, cortical involvement (V7); f22, non-enhancing tumor crosses midline (V8); f23, enhancing tumor crosses midline (V9); and T2-FLAIR mismatch (V10).

### Classification Performance

**Figure 2:**
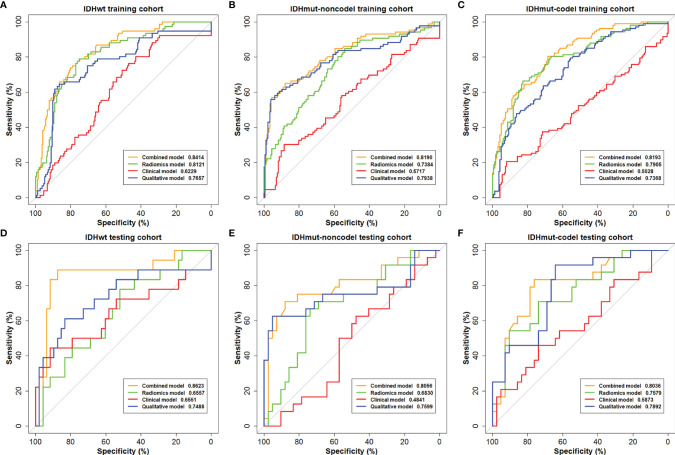
The receiving operating characteristics (ROC) curves of the combined model, the radiomics model, the clinical model, and the qualitative model on the **(A–C)** training cohort and **(D–F)** testing cohort.

The ROC curves of the combined model, radiomics model, clinical model, and qualitative model for the training and testing cohorts are shown in **Figure 2**. The AUC of the radiomics model was 0.6557 for IDHwt, 0.6830 for IDHmut-noncodel, and 0.72579 for IDHmut-codel in the testing cohort. When combining the radiomic features with the qualitative features and clinical factors, the AUCs of the combined model were 0.8623 for IDHwt, 0.8056 for IDHmut-noncodel, and 0.8036 for IDHmut-codel, in the testing cohort, with balanced accuracies of 0.8924, 0.8066, and 0.8095, respectively. Significant differences in AUCs between the radiomics model and the combined model were found for IDHmut-noncodel and IDHmut-codel in the training and testing cohorts (DeLong p < 0.05). The AUCs of the clinical model in the testing cohort were 0.6551 for IDHwt, 0.4841 for IDHmut-noncodel, and 0.5873 for IDHmut-codel. In the qualitative model, the AUCs in the testing cohort were 0.7488 for IDHwt, 0.7599 for IDHmut-noncodel, and 0.7892 for IDHmut-codel. The AUCs of the radiomics model without ADC sequence in the testing cohort were 0.7234 for IDHwt, 0.5144 for IDHmut-noncodel, and 0.6533 for IDHmut-codel, which have significant differences compared with the radiomics model for IDHmut-noncodel and IDHmut-codel (DeLong p < 0.05). The classification performance of the radiomics model and the combined model in both the training and testing cohorts are summarized in **Tables 3** and **4**, respectively. The performance of the clinical, qualitative, and no ADC sequence radiomics models is shown in **Supplementary Tables S3–S5**, respectively.

**Table 3:** Summary of the subtype-specific classification performance of the radiomics model.

Molecular subgroups	Cohorts	AUC	BAL_ACC	SEN	SPE
IDHwt	Training	0.8121 (0.7559-0.8682)	0.7782 (0.6989-0.8401)	0.7895 (0.6444-0.8947)	0.7668 (0.6528-0.8912)
	Testing	0.6557 (0.5084-0.8029)	0.6806 (0.4394-0.8181)	0.7778 (0.2778-100.00)	0.5833 (0.2292-0.9792)
IDHmt-noncodel	Training	0.7384 (0.6739-0.8030)	0.7052 (0.5462-0.7510)	0.8256 (0.5462-0.9186)	0.5847 (0.4699-0.8306)
	Testing	0.6830 (0.5478-0.8183)	0.7232 (0.5758-0.8333)	0.7083 (0.5000-0.9177)	0.7381 (0.3810-0.8810)
IDHmt-codel	Training	0.7905 (0.7351-0.8459)	0.7595 (0.6989-0.8104)	0.7103 (0.5888-0.8598)	0.8086 (0.6420-0.8827)
	Testing	0.7579 (0.6359-0.8799)	0.7500 (0.6212-0.8636)	0.6667 (0.4583-0.9594)	0.8333 (0.4524-0.9762)

BAL_ACC, SEN, and SPE are short for balanced accuracy, sensitivity, and specificity, respectively. The 95% confidence interval for each index is shown.

**Table 4:** Summary of the subtype-specific classification performance of the combined model.

Molecular subtypes	Cohorts	AUC	BAL_ACC	SEN	SPE
IDHwt	Training	0.8414 (0.7906-0.8922)	0.7911 (0.6876-0.8476)	0.7895 (0.6184-0.9342)	0.7927 (0.6062-0.9119)
	Testing	0.8623 (0.7453-0.9792)	0.8924 (0.8182-0.9697)	0.8889 (0.7222-1.0000)	0.8958 (0.7917-0.9792)
IDHmt-noncodel	Training	0.8190 (0.7604-0.8776)	0.7761 (0.7212-0.8699)	0.6395 (0.5000-0.8488)	0.9126 (0.6632-0.9781)
	Testing	0.8056 (0.6833-0.9278)	0.8066 (0.7424-0.9091)	0.7083 (0.5000-0.9167)	0.9048 (0.7381-1.0000)
IDHmt-codel	Training	0.8193 (0.7695-0.8692)	0.7552 (0.6877-0.8104)	0.7944 (0.5514-0.9159)	0.7160 (0.5802-0.9198)
	Testing	0.8036 (0.6898-0.9173)	0.8095 (0.7121-0.8939)	0.8333 (0.5833-0.9583)	0.7857 (0.6667-0.9524)

BAL_ACC, SEN, and SPE are short for balanced accuracy, sensitivity, and specificity, respectively. The 95% confidence interval for each index is shown.

**Figure 3:**
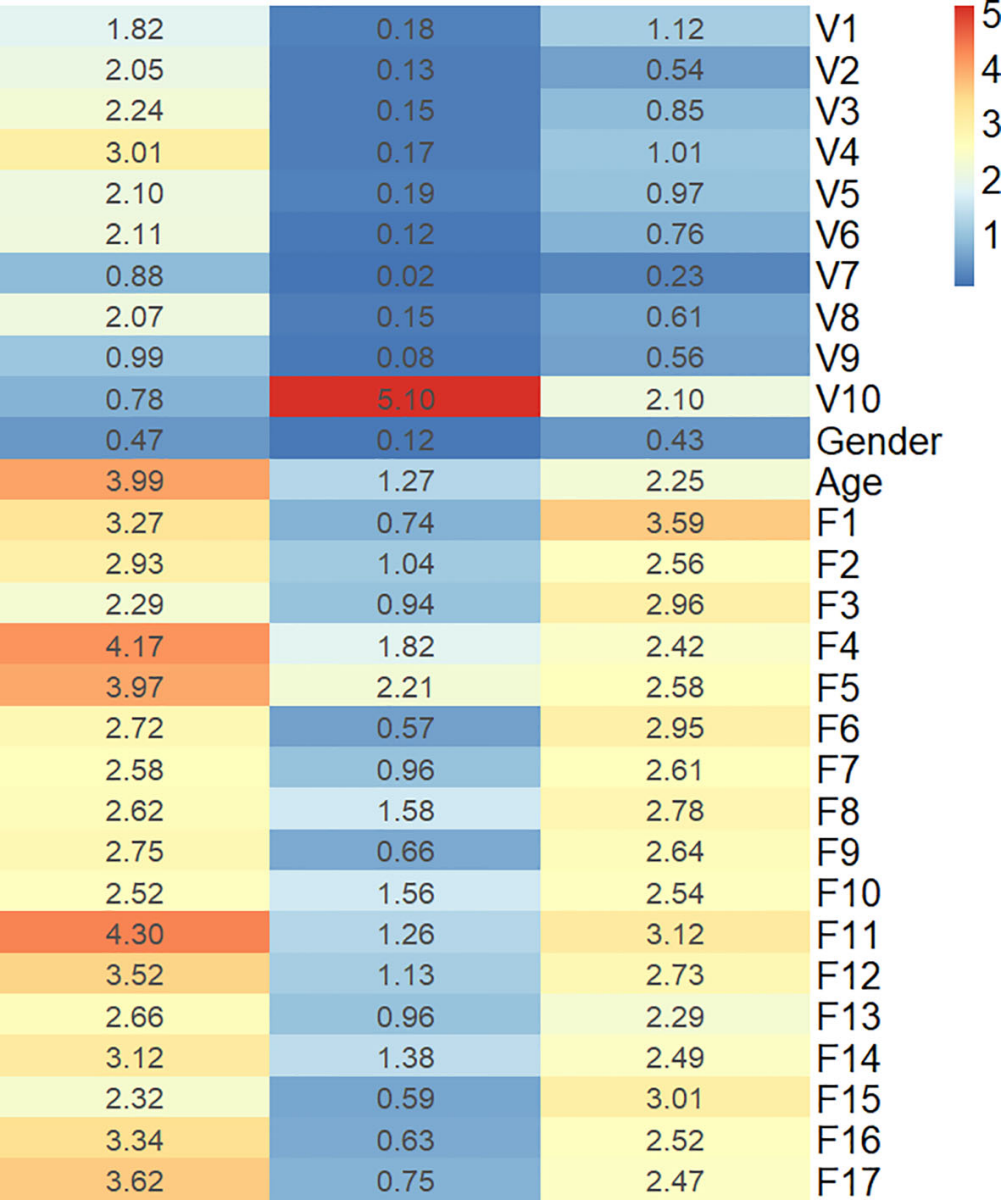
Heat map of the subtype-specific importance of all features used in subtype classification.

To describe the univariate contribution of each parameter used for subtype classification, a heat map of the subtype-specific parameter importance in the classification is shown in **Figure 3**. The meanings of the 17 radiomics features are detailed in **Supplementary Table S6**. The Gini index was calculated as the importance value for building the combined model, indicating the univariate contribution to the classification. A larger value indicates greater importance in classifying a specific subgroup.

## Discussion

In this study, we built a combined model using data from multiparametric MRI radiomics, qualitative features (VASARI scoring system and T2-FLAIR mismatch signs), and clinical factors (gender and age), which showed high feasibility for prediction of the molecular subtypes in LGG. As so, compared with the independent radiomics, clinical, and qualitative models, the combined model could be the most promising, and in the current study, we showed several of its advantages. First, we provided a multiclass classifier to preoperatively predict the molecular subtypes of LGG with satisfactory performance. This method seems to be convenient and eligible for rapid diagnosis, and it provides more guidance for clinical decisions. Second, qualitative MRI features and radiomics features were combined to obtain better predictive performance than when using an independent set of features. Third, T1WI, CE-T1WI, T2WI, FLAIR, and ADC MR sequences have been used to extract radiomic signatures, which are the most integrated MRI sequences to date.

In recent years, radiomics has been widely used to classify tumor phenotypes and to predict disease progression (27). Previous machine learning-based studies have shown the significance of radiomics in predicting molecular markers for LGG (17, 18, 28). However, these studies extracted radiomic features from conventional MRI only. DWI-derived ADC imaging is a measure of the magnitude of diffusion of water molecules within a tissue. Maynard et al. reported that ADC parameters enabled molecular subtypes of LGG discrimination (19). In the present study, we achieved good performance for predicting IDHwt and IDHmut-codel after incorporating the ADC information into the radiomics model. Lu et al. created a three-level binary classification model to predict the glioma subtypes (16). The binary classifier of 1p19q status was applied to the classified IDH-mutation LGG subtype; hence, predicting subtypes of LGG still required two separate classifications, which may increase the difficulty of clinical application. The multiclassification model in our study offers a more convenient method for predicting the subtypes of LGG.

Although the evaluation of qualitative radiographic features has certain limitations, visually accessible features still have good performance in predicting molecular markers. Zhou et al. identified 165 LGG patients through VASARI annotations, which reached AUCs of 0.73 for the IDH mutation and 0.78 for the 1q19q codeletion prediction (10). Park et al. built a model using the VASARI dataset to predict IDH mutation status in LGG; the AUCs were 0.859 and 0.788 in the discovery and validation sets, respectively (29). The T2-FLAIR mismatch sign was also considered an important imaging biomarker for predicting IDH and 1p19q status in LGG (12). Broen et al. confirmed the 100% positive predictive value (PPV) for the T2-FLAIR mismatch sign in predicting IDHmut-noncodel astrocytoma in a multi-institution cohort of LGG (30). Moreover, recent studies have shown that IDH mutation status is related to the T2-FLAIR mismatch sign (31, 32). Therefore, the T2-FLAIR sign was included in the present study. The qualitative model in our study achieved an acceptable performance. In addition, the average age of patients with IDHwt gliomas is several years higher than that of patients with IDH-mutated gliomas (10, 33). Gender was also included as a routine and easily accessible clinical factor. Thus, a better classification performance is expected from a combined radiomics, qualitative, and clinical model.

Recently, Zhou et al. extracted radiomic features from CE-T1WI, T2WI, and FLAIR sequences to develop two separate predictive models for IDH and 1p19q status of LGG (17). Their models achieved acceptable performance for predicting IDH mutants but general performance for predicting 1p19q status with an AUC of 0.685–0.716. The reasons for the general performance on predicting the 1p19q status might be related to the limited MR sequences, the relatively small sample size, and the lack of qualitative features. In our study, we achieved better performance with AUCs of 0.8056 and 0.8036 for IDHmut-noncodel and IDHmut-codel, respectively.

The combined model in the current study demonstrated better performance in differentiating LGG subtypes than any other independent model, with AUCs of 0.8623, 0.8056, and 0.8036 for IDHwt, IDHmut-noncodel, and IDHmut-codel in the testing cohort, respectively. The heat map of feature importance in **Figure 3** shows that the T2-FLAIR mismatch sign contributes the most for predicting the IDHmut-noncodel subtype. Eleven out of the 17 selected radiomic features were texture features. This is consistent with the conclusions of previous studies, in which texture measurements describing spatial variations of tumor intensity were the most illustrative for the IDH and 1p19q genotypes (16).

A recent study reported that the majority of IDHwt LGGs were underdiagnosed as glioblastomas (GBMs) (34). The outcome of IDHwt LGG has been shown to be indistinguishable from that of IDHwt GBM and worse than that of IDH mutant GBM (35). The 2021 WHO classification of CNS tumors classifies adult-type diffuse gliomas into three subtypes: IDH-mutant astrocytoma, IDH-mutant and 1p19q-codeleted oligodendroglioma, and IDHwt GBM (4). Our model is also suitable for this classification method.

The current study has several limitations. First, this was a retrospective study in which all enrolled patients were from a single hospital. Further prospective multicenter studies are needed. Second, although we have reviewed all conventional MRI sequences and DWI sequences, some advanced MRI sequences [e.g., magnetic resonance spectroscopy (MRS), diffusion tensor imaging, perfusion-weighted imaging] could reflect the microstructure and metabolic information of tumors and improve the prediction performance for IDH mutation and 1p19q status. It has been reported that a classifier based on MRS can discriminate IDH mutation status with satisfactory performance (36).

In conclusion, we developed an efficient machine learning-based combined model with reliable performance for predicting the molecular subtypes of LGG. Our model may have the potential to serve as a non-invasive tool to complement invasive tissue sampling and guide the individualized management of patients with LGG.

## Data Availability Statement

The original contributions presented in the study are included in the article/**Supplementary Material**. Further inquiries can be directed to the corresponding authors.

## Ethics Statement

The studies involving human participants were reviewed and approved by Human Scientific Ethics Committee of the First Affiliated Hospital of Zhengzhou University (No. 2019-KY-176). Written informed consent to participate in this study was provided by the participants’ legal guardian/next of kin.

## Author Contributions

JY, ZZ, and XL conceptualized and designed the study. CS, LF, WQW, WWW, WD, TS, HZ, YZ, DP, ZL, XH, XW, WL, and YG acquired the data. CS, LL, ZCL, JC, and YW analyzed and interpreted the data. CS and LF drafted the manuscript. JY and ZZ critically revised the manuscript. All authors contributed to the article and approved the submitted version.

## Funding

This research was supported by the National Natural Science Foundation of China (No. 82102149, U20A20171, 61901458, 61571432, 81702465, 8217111948, 82173090, U1804172, U1904148), the Science and Technology Program of Henan Province (No. 202102310136, 202102310138, 202102310113, 202102310083), The Key Program of Medical Science and Technique Foundation of Henan Province (No. SBGJ202002062), The Joint Construction Program of Medical Science and Technique Foundation of Henan Province (No. LHGJ20190156), and Youth Innovation Promotion Association of the Chinese Academy of Sciences (2018364).

## Conflict of Interest

The authors declare that the research was conducted in the absence of any commercial or financial relationships that could be construed as a potential conflict of interest.

## Publisher’s Note

All claims expressed in this article are solely those of the authors and do not necessarily represent those of their affiliated organizations, or those of the publisher, the editors and the reviewers. Any product that may be evaluated in this article, or claim that may be made by its manufacturer, is not guaranteed or endorsed by the publisher.
