# Supplementary material for: Radiomics and Qualitative Features From Multiparametric MRI Predict Molecular Subtypes in Patients With Lower-Grade Glioma
Source: Front Oncol. 2022 Jan 21;11:756828. doi: 10.3389/fonc.2021.756828 (PMC8814098; doi:10.3389/fonc.2021.756828)

**Supplementary Material**

**MR machines and sequence parameters**

The 1.5 T scanners included Siemens Healthcare (Magnetom Avanto/Aera, Erlangen, Germany), and Philips Healthcare (Achieva, Best, Netherlands). The 3.0 T scanners included Siemens Healthcare (Magnetom Skyra/Prisma/Trio TIM/verio, Erlangen, Germany), GE Healthcare (Discovery MR750/MR750w/Signa HDxt, Milwaukee, WI, USA), and Philips Healthcare (Ingenia, Best, Netherlands).

T1WI and CE-T1WI: repetition time (TR), 163-3019 ms; echo time (TE), 2.03-43 ms; echo train length (ETL), 1-19; section thickness, 5-6 mm; image slice spacing, 1-2 mm; number of averages/excitations, 1-3; flip angle (FA), 45°-111°; field of view (FOV), 220×206-240×240 mm^2^; matrix, 184×184-320×320 mm^2^.

T2WI: TR, 1090-9186 ms; TE, 70-130 ms; ETL, 16-32; section thickness, 5-6 mm; image slice spacing, 1-2 mm; number of averages/excitations, 1-2; FA, 90°-150°; FOV, 220×184-240×240 mm^2^; matrix, 220×220-512×512 mm^2^.

FLAIR: TR, 4000-9000 ms; TE, 79-174 ms; inversion time (TI), 1530-2500 ms; ETL, 1-38; section thickness, 5-6 mm; image slice spacing, 1-2 mm; number of averages/excitations, 1-2; FA, 90°-160°; FOV, 220×193-240×240 mm^2^; matrix, 176×176-320×224 mm^2^.

DWI: All DWI acquisitions were acquired before injection of a 0.1 mmol/kg dose of gadolinium-based contrast agent (Gadopentetic Acid Dimeglumine Salt Injection, Magnevist, Bayer Healthcare, Berlin, Germany, or Gadoteric Acid Meglumine Salt Injection, Hengrui Healthcare, Jiangsu, China), and were used a spin echo single-shot echo-planar sequence including the values of b = 0 and b = 1000 s/mm^2^, with diffusion sensitizing gradients encoded in the x, y, and z directions. The sequence parameters were TR, 2121-6000 ms; TE, 77-119 ms; ETL, 1-82; section thickness, 5 mm; image slice spacing, 1.5 mm; number of averages/excitations, 1; FA, 90°; FOV, 220×220-240×240 mm^2^; matrix, 152×114-192×192 mm^2^. The corresponding ADC maps were generated with a monoexponential model on a voxel-by-voxel basis for all imaging planes using the Syngo workstation (Siemens Healthcare, Erlangen, Germany), or the Advantage workstation (Version 4.6, GE Healthcare, Milwaukee, WI, USA), or the workstation (Philips Healthcare, Best, Netherlands).

**Supplementary Table 1** A summary of the radiomics features extracted.

Fourteen shape features describing the 3D geometric characteristics of the VOI were extracted. From the original images and the transformed images (applying wavelet transform or LoG filtering), 234 intensity features describing the first-order distribution of the intensities were extracted, while 949 texture features were computed to describe the patterns, or the high-order intensity distributions with five methods, including gray-level co-occurrence matrix (GLCM), gray-level run length matrix (GLRLM), gray level size zone matrix (GLSZM), gray level dependence matrix (GLDM), and neighborhood gray-tone difference matrix (NGTDM). Totally, 5929 features were extracted from five MR sequences.

| **Feature Classes** | | **Feature Names** |
| --- | --- | --- |
| Shape Features | | Elongation, Flatness, Least Axis Length, Major Axis Length, Maximum 2D Diameter Column, Maximum 2D Diameter Row, Maximum 2D Diameter Slice, Maximum 3D Diameter, Mesh Volume, Minor Axis Length, Sphericity, Surface Area, Surface Volume Ratio, Voxel Volume |
| Intensity Features | | Maximum, Median, Minimum, Mean, Energy, Entropy, Variance, Kurtosis, Root Mean Square, Skewness, 10th Percentile, 90th Percentile, Mean Absolute Deviation, Uniformity, Range, Robust Mean Absolute Deviation, Total Energy, Interquartile Range |
| Texture  Features | GLCM  Features | Contrast, Correlation, Autocorrelation, Cluster Tendency, Sum Average, Sum Entropy, Sum Squares, Difference Average, Difference Variance, Difference Entropy, Cluster Prominence, Cluster Shade, Maximum Probability, Inverse Difference Moment, Informational Measure of Correlation 1/2, Inverse Difference Moment Normalized, Inverse Difference Normalized, Inverse Difference, Inverse Variance, Maximal Correlation Coefficient, Joint Average, Joint Energy, Joint Entropy |
|  | GLDM  Features | Dependence Entropy, Dependence Non-Uniformity, Dependence Non-Uniformity Normalized, Dependence Variance, Gray-Level Non-Uniformity, Gray-Level Variance, High Gray-Level Emphasis, Large Dependence Emphasis, Large Dependence High Gray-Level Emphasis, Large Dependence Low Gray-Level Emphasis, Low Gray-Level Emphasis, Small Dependence Emphasis, Small Dependence High Gray-Level Emphasis, Small Dependence Low Gray-Level Emphasis |
|  | GLRLM  Features | Gray-Level Non-uniformity, Gray-Level Non-uniformity Normalized, Gray-Level Variance, High Gray-Level Run Emphasis, Long Run Emphasis, Long Run High Gray-Level Emphasis, Long Run Low Gray-Level Emphasis, Low Gray-Level Run Emphasis, Run Entropy, Run Length Non-Uniformity, Run Length Non-Uniformity Normalized, Run Percentage, Run Variance, Short Run Emphasis, Short Run High Gray-Level Emphasis, Short Run Low Gray-Level Emphasis |
|  | GLSZM  Features | Gray-Level Non-Uniformity, Gray-Level Non-Uniformity Normalized, Gray-Level Non-Uniformity Normalized, High Gray-Level Zone Emphasis, Large Area Emphasis, Large Area High Gray-Level Emphasis, Large Area Low Gray-Level Emphasis, Low Gray-Level Zone Emphasis, Size Zone Non-Uniformity, Size Zone Non-Uniformity Normalized, Small Area Emphasis, Small Area High Gray-Level Emphasis, Small Area Low Gray-Level Emphasis, Zone Entropy, Zone Percentage, Zone Variance |
|  | NGTDM  Features | Coarseness, Contrast, Busyness, Complexity, Strength |

**Supplementary Table 2** Distribution of patient characteristics across the three molecular subtypes.

| **Characteristic** | **IDHwt (n=94)** | **IDHmut-noncodel (n=110)** | **IDHmut-codel (n=131)** | ***P* Value** |
| --- | --- | --- | --- | --- |
| Gender |  |  |  | 0.3755 |
| Male | 50 (53.19%) | 68 (61.82%) | 71 (54.20%) |  |
| Female | 44 (46.81%) | 42 (38.18%) | 60 (45.80%) |  |
| Age(year)* | 49.22±15.39 | 41.26±10.52 | 44.92±10.60 | <0.0001 |

Note. Unless otherwise noted, data are numbers of patients, with percentages in parentheses.
*Data are means ± standard deviations.

**Supplementary Table 3** Summary of the subtype-specific classification performance of the clinical model.

| **Molecular subgroups** | **Cohorts** | **AUC** | **BAL_ACC** | **SEN** | **SPE** |
| --- | --- | --- | --- | --- | --- |
| IDHwt | Training | 0.6229  (0.5529-0.6929) | 0.6423  (0.4535-0.6580) | 0.8026  (0.5789-0.9605) | 0.4819  (0.2746-0.6684) |
|  | Testing | 0.6551  (0.4840-0.8262) | 0.7084  (0.5909-0.8788) | 0.5000  (0.2222-0.8889) | 0.9167  (0.5208-1.0000) |
| IDHmt-noncodel | Training | 0.5717  (0.4964-0.6471) | 0.6027  (0.5166-0.6952) | 0.3256  (0.2209-0.7907) | 0.8798  (0.4044-0.9344) |
|  | Testing | 0.4841  (0.3396-0.6286) | 0.5923  (0.3788-0.6818) | 0.7083  (0.1250-1.0000) | 0.4762  (0.0238-0.9524) |
| IDHmt-codel | Training | 0.5028  (0.4297-0.5759) | 0.5690  (0.5613-0.6929) | 0.2243  (0.1402-0.5888) | 0.9136  (0.5554-0.9568) |
|  | Testing | 0.5873  (0.4390-0.7356) | 0.6518  (0.4545-0.7576) | 0.5417  (0.1667-1.0000) | 0.7619  (0.1429-1.0000) |

BAL_ACC, SEN and SPE were short for balanced accuracy, sensitivity, and specificity, respectively. The 95% confidence interval for each index was shown.

**Supplementary Table 4** Summary of the subtype-specific classification performance of the qualitative model.

| **Molecular subtypes** | **Cohorts** | **AUC** | **BAL_ACC** | **SEN** | **SPE** |
| --- | --- | --- | --- | --- | --- |
| IDHwt | Training | 0.7657  (0.7008-0.8306) | 0.7681  (0.7100-0.8587) | 0.6579  (0.5395-0.8289) | 0.8782  (0.6788-0.9275) |
|  | Testing | 0.7488  (0.6029-0.8948) | 0.7674  (0.5909-0.8788) | 0.7222  (0.3889-0.9444) | 0.8125  (0.4792-1.0000) |
| IDHmt-noncodel | Training | 0.7938  (0.7288-0.8589) | 0.7754  (0.7508-0.8699) | 0.6163  (0.5000-0.7794) | 0.9344  (0.7432-0.9836) |
|  | Testing | 0.7599  (0.6178-0.9020) | 0.7887  (0.7424-0.9091) | 0.6250  (0.4167-0.8333) | 0.9524  (0.7619-0.1000) |
| IDHmt-codel | Training | 0.7368  (0.6769-0.7967) | 0.6978  (0.6208-0.7584) | 0.7009  (0.4299-0.9065) | 0.6944  (0.4568-0.9136) |
|  | Testing | 0.7892  (0.6779-0.9005) | 0.7917  (0.6515-0.8485) | 0.9167  (0.5833-1.0000) | 0.6667  (0.5000-0.9286) |

BAL_ACC, SEN and SPE were short for balanced accuracy, sensitivity, and specificity, respectively. The 95% confidence interval for each index was shown.

**Supplementary Table 5** Summary of the subtype-specific classification performance of the radiomics model without ADC sequence.

| **Molecular subtypes** | **Cohorts** | **AUC** | **BAL_ACC** | **SEN** | **SPE** |
| --- | --- | --- | --- | --- | --- |
| IDHwt | Training | 0.7729  (0.7105-0.8352) | 0.7399  (0.6208-0.8067) | 0.6974  (0.5658-0.9211) | 0.7824  (0.5077-0.8653) |
|  | Testing | 0.7234  (0.5866-0.8601) | 0.7465  (0.5303-0.8636) | 0.7222  (0.5000-1.0000) | 0.7708  (0.3958-0.9167) |
| IDHmt-noncodel | Training | 0.6612  (0.5898-0.7326) | 0.6549  (0.5799-0.7509) | 0.6977  (0.3023-0.8023) | 0.6120  (0.5191-0.9563) |
|  | Testing | 0.5144  (0.3606-0.6681) | 0.6101  (0.4242-0.7424) | 0.4583  (0.1250-0.9583) | 0.7619  (0.1429-0.9762) |
| IDHmt-codel | Training | 0.7573  (0.7002-0.8144) | 0.7135  (0.6283-0.7658) | 0.7664  (0.5047-0.9346) | 0.6605  (0.4506-0.8889) |
|  | Testing | 0.6533  (0.5093-0.7973) | 0.6994  (0.5303-0.8030) | 0.5417  (0.2500-0.9583) | 0.8571  (0.3095-0.9762) |

BAL_ACC, SEN and SPE were short for balanced accuracy, sensitivity, and specificity, respectively. The 95% confidence interval for each index was shown.

**Supplementary Table 6** The meanings of the 17 radiomics features constituting the radiomics model.

To fully characterize the image phenotypes within the tumor, we extracted radiomics features from not only the original medical images but also the transformed, or derived images by using wavelet or Laplacian of Gaussian (LoG) filters onto the original images. Finally, we extracted radiomics features from three types of images: original images, wavelet images, and LoG images. Wavelet images were obtained by applying wavelet transform on the original images. Wavelet transform can decouple informative textures by decomposing the original images into multiple low- and high-frequency components. Let *H* and *L* be a high-pass and low-pass wavelet function, respectively. Then, the eight decomposed images can be denoted as ***I****_HHH_*, ***I****_HHL_*, ***I****_HLH_*, ***I****_HLL_*, ***I****_LHH_*, ***I****_LHL_*, ***I****_LLH_*, ***I****_LLL_*, where the three subscripts meant the high- or low-pass filtering operations along *x*, *y* and *z* directions of the original 3D MR image. LoG images were obtained by applying LoG filtering operation on the original images. LoG performs two filtering operations, a Gaussian filtering, and a Laplacian filtering. Finally, 17 features comprising 11 texture features and 6 intensity features were selected for the final radiomics model building. All 17 features were shown in **Table 2**. The meanings of these features are described as follows.

| **No.** | **Selected Features** | **Meanings** |
| --- | --- | --- |
| *f*_1_ | InterquartileRange | interquartile range=P_75_−P_25_. P_25_ and P_75_ are the 25^th^ and 75^th^ percentile of the image array, respectively. |
| *f*_2_ | Skewness | Skewness measures the asymmetry of the distribution of values about the Mean value. *f*_2_ is extracted from ***I****_LHL_* of ADC original image. |
| *f*_3_ | NGTDM Complexity | Calculate and return the complexity. An image is considered complex when there are many primitive components in the image, i.e. the image is non-uniform and there are many rapid changes in gray level intensity. |
| *f*_4_ | GLCM ClusterShade | A measure of the image uniformity. A higher cluster shade implies greater asymmetry about the mean. |
| *f*_5_ | GLRLM RunVariance | RV is a measure of the variance in runs for the run lengths. |
| *f*_6_ | Median | The median gray level intensity within the tumor area. |
| *f*_7_ | GLCM ClusterShade | A measure of the image uniformity. A higher cluster shade implies greater asymmetry about the mean. |
| *f*_8_ | GLCM Imc1 | IMC1 assesses the correlation between the probability distributions of i and j (quantifying the complexity of the texture). |
| *f*_9_ | GLRLM RunVariance | The same as *f*_5_ but extracted from FLAIR LoG image. |
| *f*_10_ | Skewness | The same as *f*_2_ but extracted from FLAIR ***I****_LHL_* image. |
| *f*_11_ | GLRLM GrayLevelNonUniformityNormalized | GrayLevelNonUniformityNormalized (GLNN) measures the similarity of gray-level intensity values in the image, where a lower GLNN value correlates with a greater similarity in intensity values. |
| *f*_12_ | GLRLM RunVariance | RV is a measure of the variance in runs for the run lengths. |
| *f*_13_ | GLCM SumEntropy | Sum Entropy is a sum of neighborhood intensity value differences. |
| *f*_14_ | GLDM LargeDependenceEmphasis | A measure of the distribution of large dependencies, with a greater value indicative of larger dependence and more homogeneous textures. |
| *f*_15_ | Skewness | The same as *f*_2_ but extracted from T1WI ***I****_LLL_* image. |
| *f*_16_ | GLRLM LongRunHighGrayLevelEmphasi | LongRunHighGrayLevelEmphasi (LRHGLRE) measures the joint distribution of long run lengths with higher gray-level values. |
| *f*_17_ | Skewness | The same as *f*_2_ but extracted from CE-T1WI original image. |

**Supplementary Figure 1** The patient selection procedure in this study.


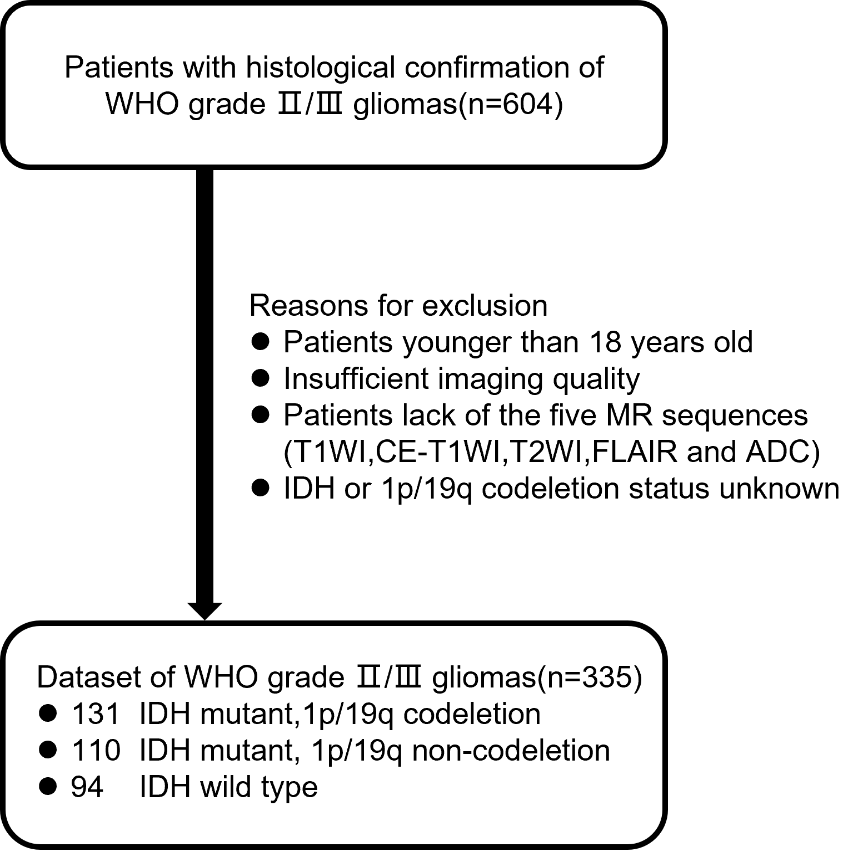


**Supplementary Figure 2** The heatmaps of the correlation coefficients of the imaging features.

The correlation coefficients were normalized to their z-scores (zero mean and unit variance) and then were clustered. The brighter the blue (red) color, the higher (lower) the correlation of a feature pair. The heatmaps showed that the correlations between feature pairs were reduced after applying the redundancy removal.


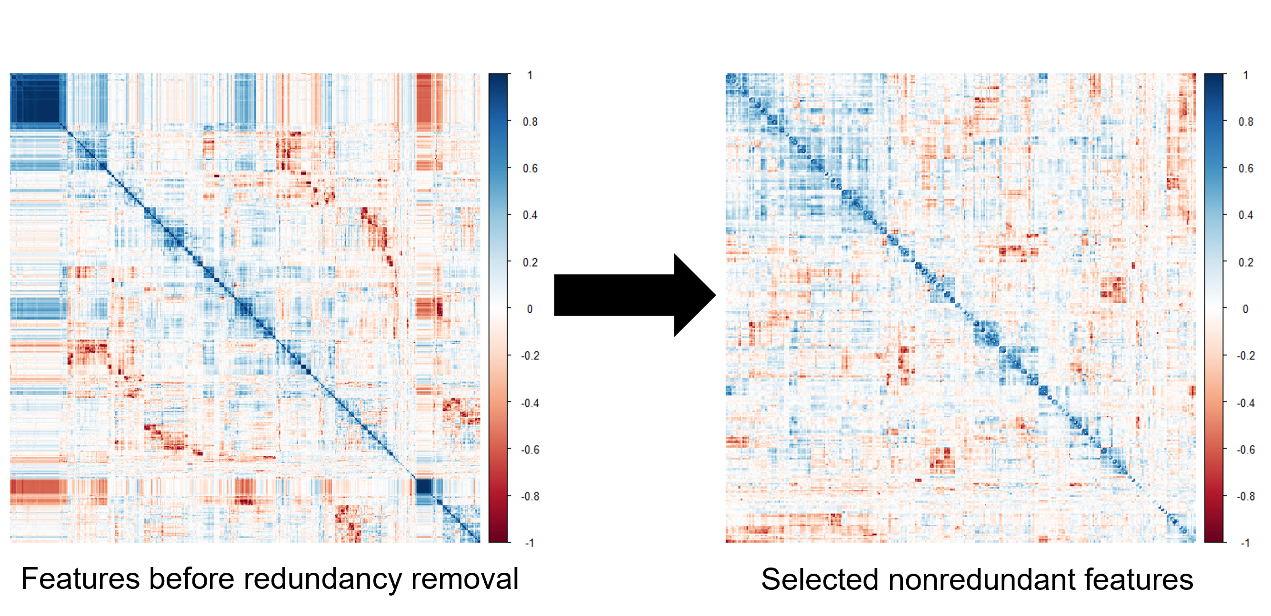


**Supplementary Figure 3** The result of the Boruta feature selection of radiomics features.

The y-axis represents the box plots of the feature importance generated by the Boruta algorithm, while the x-axis represents all 335 features fed to the Boruta algorithm. Finally, 17 features with largest importance values were selected, as shown in green. The discarded features with smaller importance values were shown in red.


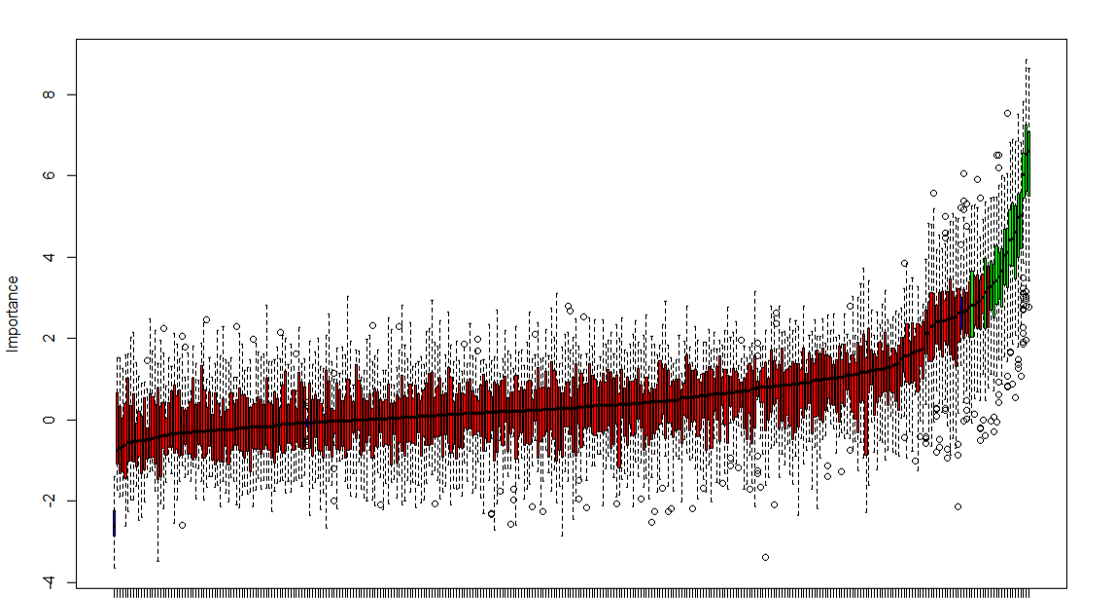


**Supplementary Figure 4** The boxplots of 17 selected radiomics features for different scanners.

The y-axis represents the box plots of the values of radiomics features, while the x-axis represents 3 different manufacturers of scanners.


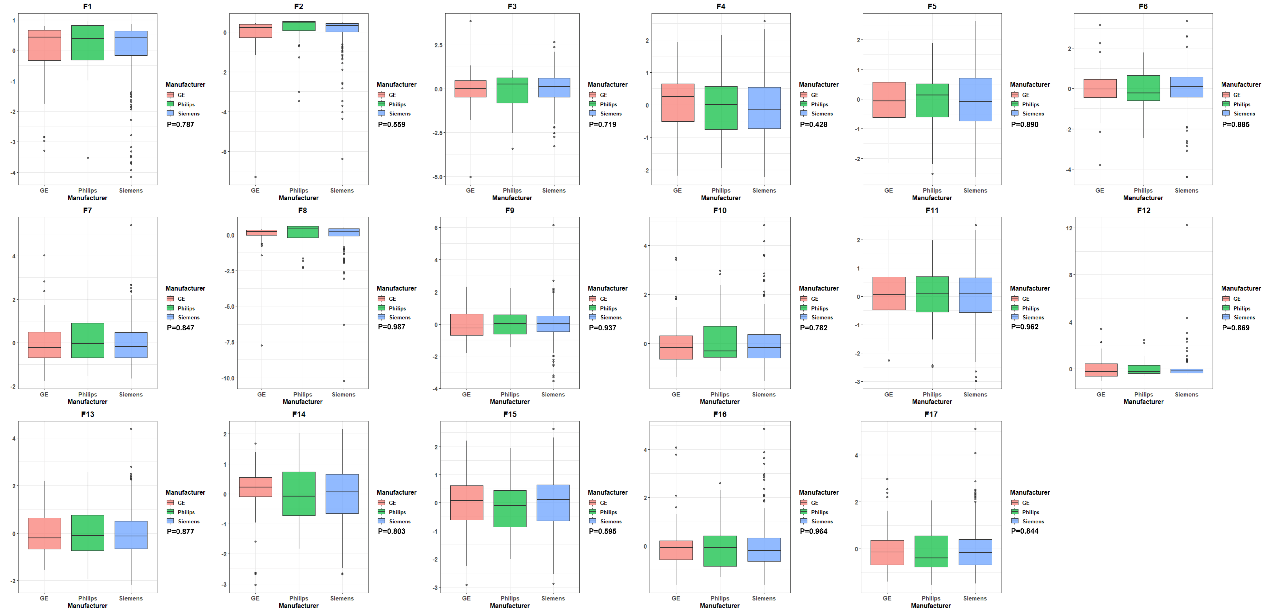


**Supplementary Figure 5** The boxplots of 17 selected radiomics features for different field strengths.

The y-axis represents the box plots of the values of radiomics features, while the x-axis represents 2 different field strengths.


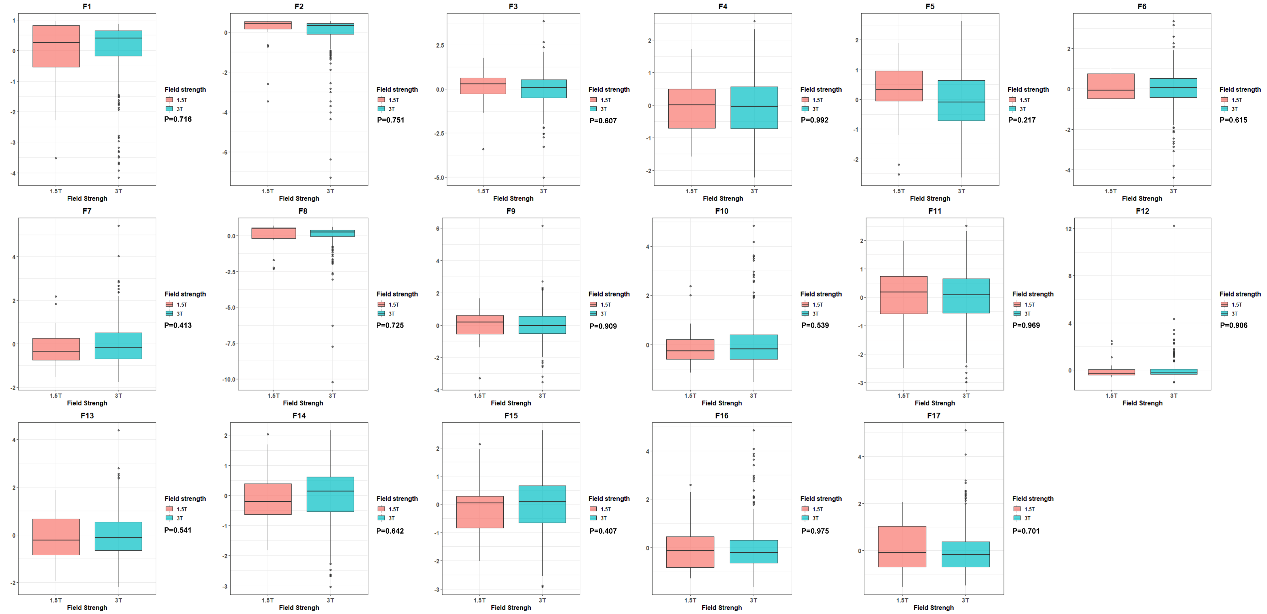


**Supplementary Figure 6** The result of the Boruta feature selection of qualitative features.

The y-axis represents the box plots of the feature importance generated by the Boruta algorithm, while the x-axis represents all 26 features fed to the Boruta algorithm. Finally, 10 features with largest importance values were selected, as shown in green. The discarded features with smaller importance values were shown in red.


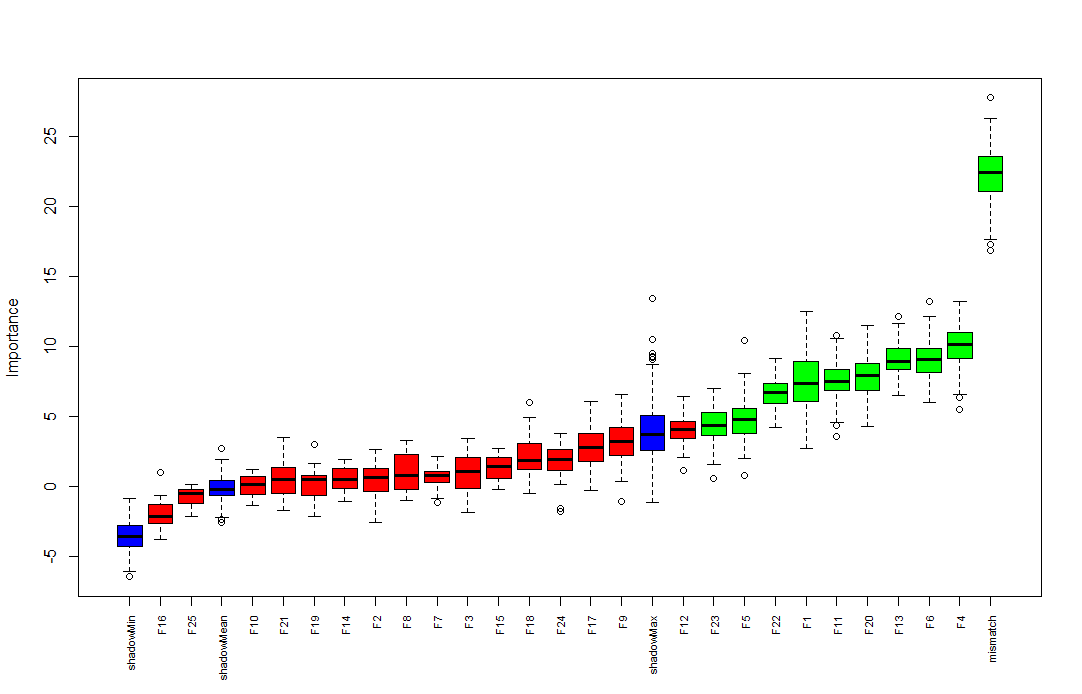

Supplement: Supplementary file 1 [file DataSheet_1.docx]
